# 

**Published:** 1878-02

**Authors:** 


*CONTENTS.*

| | Page |
|---|---|
| Poetry, Music, and Health | 375 |
| Jacob's Dream | 381 |
| Frosty Weather | 382 |
| Life Lengthened | 382 |
| Outbursts of Passion | 383 |
| Sea Sickness | 383 |
| Sea Voyages | 384 |
| Horseback Journey | 385 |
| Protecting the Feet | 385 |
| Pure Air on the Sea | 385 |
| Glycerine | 386 |
| Mineral Waters | 388 |
| Ingrowing Nails | 389 |
| Shoes | 389 |
| Sometimes | 391 |
| Hands | 392 |
| Corns | 393 |
| Warts | 393 |
| Dentifrices | 395 |
| A Cup of Tea | 396 |
| Bequests to Children | 397 |
| Sleep | 397 |
| Imprudence Versus Climate | 398 |
| Ventilation | 399 |
| How to Argue | 399 |
| "Not Dead, but Risen" (Poetry) | 400 |

